# Network Analysis of Academic Medical Center Websites in the United States

**DOI:** 10.1038/s41597-023-02104-3

**Published:** 2023-04-28

**Authors:** Shuhan He, David Chen, Kameron Collin Black, Paul Chong, Sammer Marzouk, Byung-Jun Yoon, Kendrick Davis, Jarone Lee

**Affiliations:** 1 Massachusetts General Hospital, Boston, USA; 2 Temerty Faculty of Medicine, University of Toronto, Toronto, Canada; 3 Oregon Health & Science University, Portland, USA; 4 Campbell University School of Osteopathic Medicine, Lillington, USA; 5 Harvard Department of Chemistry and Chemical Biology, Cambridge, USA; 6 Texas A&M University, College Station, USA; 7 Brookhaven National Laboratory, Upton, USA; 8 University of California, Riverside, USA

**Keywords:** Health services, Network topology, Data processing

## Abstract

Healthcare resources are published annually in repositories such as the AHA Annual Survey Database^TM^. However, these data repositories are created via manual surveying techniques which are cumbersome in collection and not updated as frequently as website information of the respective hospital systems represented. Also, this resource is not widely available to patients in an easy-to-use format. Network analysis techniques have the potential to create topological maps which serve to aid in pathfinding for patients in their search for healthcare services. This study explores the topological structure of forty United States academic health center websites. Network analysis is utilized to analyze and visualize 48,686 webpages. Several elements of network structure are examined including basic network properties, and centrality measures distributions. The Louvain community detection algorithm is used to examine the extent to which these techniques allow identification of healthcare resources within networks. The results indicate that websites with related healthcare services tend to form observable clusters useful in mapping key resources within a hospital system.

## Background & Summary

Network analysis (NA) is an increasingly important means of visualizing digital information. NA is defined as the set of techniques that are used to depict and analyze the relationships and interactions among actors and to analyze the social structures that come from the recurrence of these relationships^1^. NA can aid in the process of describing systems composed of non-identical elements that have non-local and complex interactions. NA also allows further understanding of interactions ranging from those between websites to the interactions of proteins and genes at a molecular level^2^.

Understanding the architecture of healthcare websites can allow the general public to find pertinent and salient health information, including health service offerings available from hospital networks^3^. Determining the optimal architecture of a hospital websites represented as networks can lead to the development of more intuitive paths that online users take to search for critical health information. Also, network analysis of numerous hospital websites potentially opens the door for the establishment of an automated surveillance system of healthcare services availability across the US^3^. Manual surveys (such as that provided by the AHA Annual Survey) have historically been the primary means to understanding healthcare services in the US, but the automation of this kind of survey has occurred in other industries helping performance: recent analyses from the tourism industry demonstrate that studying hyperlink connectivity can result in the increased understanding of resource availability (American Hospital Association, 2022, Annual Survey Database section)^4–6^.

The development of NA on websites first began through natural text clustering analysis, which looked at certain defined metrics and how they connect different websites on the internet; these metrics include density, betweenness centrality, and eigenvector centrality^7^. Several metrics that relate to the development of the visualization include the degrees of centrality, the betweenness centrality, the global clustering coefficient, and the average path length. Hyperlinks are quick connections between different websites along the World Wide Web (WWW)^4^. Through previous simulations, it was shown that hyperlinks provide direct connections in NA to other parts of the webspace and improve the general visibility of clustering analysis. Hyperlinks also provide another dimension of network structure to analyze to understand the connections across the WWW beyond traditional webpage content scraping^8^. Hyperlink analysis has a sparse, centralized, and hierarchical structure that may be used to group websites based on the frequency of community interactions, among other possible analyses^5^.

This study focused on hospital websites, concentrating specifically on the hyperlink parameters of the clustering coefficient, the modularity (community detection), assortativity (node similarity based on degree), and the reciprocity (tendency of node pairs to form connections with each other). This analysis has since been corroborated by additional analyses by other organizations which look at the assortativity of hyperlinks and link strength^6,9^.

We propose the creation of a novel healthcare website analysis data repository in which healthcare organizations would have their NA information collected in order to map the web structure and hyperlink activity between the organizations^10^. This would have the possibility of improving website functionality by (1) identifying key features of the website structure to characterize how users navigate between different webpages and (2) automate visualization of the healthcare services offered by different AMCs (AMC). To address the first aim, the proposed dataset contains the nodes and edges of webpages and hyperlinks between webpages respectively for different AMCs, allowing further study of how to optimize connectivity between webpages by adding new hyperlinks so that users can find health-related information intuitively through as few links as possible. Moreover, this dataset can help medical centers strategically market the online visibility of webpages of lesser-known services by increasing their connectivity to other webpages that users often must traverse while navigating through the website. To address the second aim, the visualization of the network topology of nodes and edges that represent website structure can be used to automate visualization of the services that an AMC offers. Further research into automatic plotting of healthcare services, such as mapping the geographic locations of different healthcare resources using hyperlink networks, can help patients find local resources. Future development of strategies to increase the connectivity and visibility of websites through characterization and optimization of hyperlink network topology has potential to reduce the time and knowledge necessary to find relevant healthcare information compared to traditional approaches based on webpage content analysis alone.

## Methods

### Sample selection

This project focused on the websites of AMCs in the United States, consisting of the 73 institutions which were listed as members on the Association of Academic Health Centers (AAHC) website as of October 25, 2021 (Association of Academic Health Centers, 2021)^10^. Websites corresponding to the primary teaching hospital associated with each AMC were identified and used in our analysis; these websites were crawled with either an unlimited depth (17 out of 73 websites) or a depth limit of three (23 of 73 websites), depending upon web domain size. Websites were excluded from analysis (33 of 73 websites) for one of two reasons: one, due to excessive web domain size that yielded data exceeding the processing capacity of the data analysis and visualization tools utilized (see Supplementary Table 1) or two, due to network security that prevented website crawling (The University of Tennessee Health Science Center and University of Oklahoma Health Sciences Center). These limitations can be addressed in future works that build upon the work done in this study.

### Data collection and analysis

All network analyses took place between January 20, 2022 to February 17, 2022. A database of webpage nodes and hyperlinks between nodes for each website was built using the Screaming Frog SEO web crawler tool (Screaming Frog, 2022, SEO section). A web crawler is a program which allows the user to index content within websites for analysis. Web crawling is designed to facilitate discovery of URLs, which contrasts from web scraping in which data is extracted from websites; examples of common web crawlers include Google Search and Microsoft Bing (Google Search, 2022; Microsoft Bing, 2022). In our study, URLs were gathered via web crawling in effort to study relationships between websites and did not include the extraction of copyrighted material.

The database created in this study contained each of the top-level URLs associated with each web page in our AMC dataset.

For example, a top-level domain, corresponding to a website’s homepage, may contain the URL: www.universityhospital.org. A subdomain, or internal link, of this web page may contain information regarding team members and be associated with the URL: www.universityhospital.org/team. Other subdomains may also include topics such as care services provided, and location.

Included in this data extraction were internal links, inlinks and outlinks. Table 1 contains definitions of these terms. The internal links (i.e. subdomains) served as nodes, or endpoints in the network analysis. Inlinks are links between web pages on a single website: these serve as internal edges, or links that connect nodes inside the subgraph being analyzed. Outlinks serve as external edges, or links between nodes that are inside the subgraph and those outside.

**Table 1:** Network topology terminology and definitions.

Term	Definition
Top-level domain	corresponding to a website’s homepage. A primary domain encompassing a distinct subset of the internet (Oxford Languages, 2022).
Subdomain	a subdivision of a primary domain. Synonymous with internal link. These websites serve as nodes, or endpoints in network analyses.
Inlink	a link between web pages on a single website. A link that connects nodes inside a subgraph. Synonymous with internal edge.
Internal edge	synonymous with inlink.
Outlink	a link between nodes inside the subgraph being analyzed and those outside of it. Synonymous with external edge.
External edge	synonymous with outlink.

**Fig. 1:**
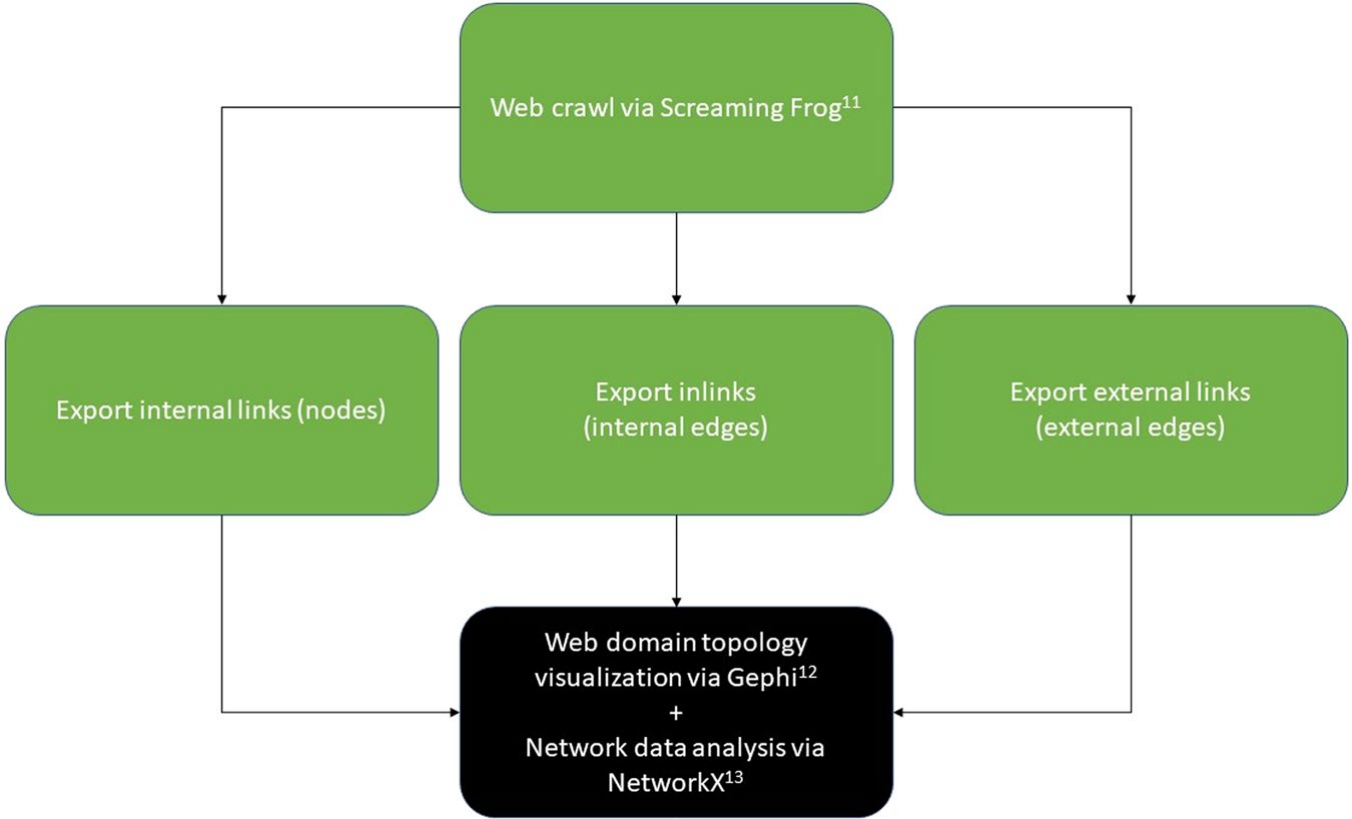
Methodology for data crawling via Screaming Frog web crawler and subsequent visualization and analysis of data via Gephi and NetworkX. The final step of data visualization/analysis included a cleaning step ridding data of URLs containing “.css” and “.js” addresses.

Each top-level URL was inputted into the Screaming Frog SEO web crawler tool with an initial unlimited crawl depth, then refined to a limited crawl depth of three as necessary to limit total crawled URLs to 3,000 or less per NetworkX/Gephi data visualization restrictions (excessively large web domains were excluded from analysis, see Limitations section and Fig. 1 for details)^10^, thereby creating a topological map of the website which included its subdomains.

Secondary processing of the data included removing edges that led to “.css” or “.js” URLs, which real users do not directly interact with and are for purely stylistic and functional purposes, respectively.

The resulting network included 48,686 nodes (web pages) and 1,165,520 edges (links) between websites. Gephi was used for network visualization purposes, general analyses, and measuring network properties^11^. For more detailed network analyses not available in Gephi, NetworkX was used, which is a Python language library package^12^. Several different network metrics were used in this study, including metrics for each node and summary metrics for the entire network. Node-specific metrics included betweenness centrality, Eigenvector, closeness, eccentricity, PageRank, and modularity. Network-wide metrics included total number of nodes, total number of edges, degree, average degree, network density, transitivity, average clustering, and diameter.

Analyses were performed at the global (network-wide) level and the individual node level. At the global level, the general structure and topology of the network was investigated. At the individual level, properties of the individual web page of one website were examined. The centrality metrics studied included betweenness, closeness, eigenvector, degree and clustering. The structural properties of each node, representing one web page, were described using the aforementioned centrality metrics. To identify the websites with the highest centrality metrics, we calculated the importance index as the normalized mean of the centrality measures. The top five AMC websites based on this metric were (in order): University of Utah Health Sciences Center, Augusta University, University of Miami, Midwestern University, and Tulane University Health Sciences Center.

### Global network-wide metrics

In order to gain a thorough understanding of the network, several network-wide analyses were conducted. These included total number of nodes, total number of edges, degree, average degree, network density, transitivity, average clustering, and diameter^5,6^. Regardless of which network property is studied, all must be examined in light of a network’s degree distribution^5^. In network analysis, the degree of a website represents the number of links the website has to other websites. In a directed network, the average node degree consists of in-degree (the number of incoming links to a website) and out-degree (the number of outgoing links from a website)^6^.

Network analysis of evolving networks has shown that many of these networks are scale-free^13^. A scale free network is a network whose degree distribution follows a power law pattern^9^. Regardless of the system and identity of its constituents, the probability P(k) that a node in a network interacts with k other nodes decays as a power law by factor of the Euler–Mascheroni constant, γ, as in Eq. (1)^2^.

1 $$P(k) \sim {k}^{-\gamma }$$

The degree distribution of a network, P_deg_(k), is defined as the fraction of the websites (or nodes, n) with degree k, as in Eq. (2).

2 $${P}_{deg}(k)=\frac{\left[n(k)\right]}{n}$$

Previous literature has described the scale-free power-law distribution of many large networks as the consequence of two generic mechanisms. One, networks expand continuously by the addition of new vertices; two, new vertices attach preferentially to sites that are already well connected^2^.

The density of a network is a metric that depicts the interconnectedness of the concepts^14^. In other words, density represents the ratio of the total number of all existing links to the total possible number of links in the network^15^. More specifically, network density is the number of links (or edges), m, among the nodes, n, divided by the number of possible links, as in Eq. (3)^14^.

3 $$Network\;density=\frac{m}{n-1}$$

Clustering is defined as a function that organizes nodes based on a proximity measure, thereby grouping similar nodes in the same cluster and dissimilar nodes in different clusters^16^. Nuances in clustering analysis have been explored in past works^16,17^.

### Individual node-specific metrics

The analysis of the network was also concentrated at the level of each individual node. Measurements applied to each node included betweenness centrality, eigenvector, closeness centrality, eccentricity, PageRank and modularity.

Fundamental to the field of social network analysis are two concepts: centrality and prestige. Studies in this field have suggested that an actor’s (or node’s) prominence in a network should be measured by its direct ties, adjacent ties, and indirect paths involving intermediary nodes^18^. More specifically, the prominent nodes in a network are the most visible and have extensive relationships with other nodes^6^. Centrality is measured in terms of closeness centrality, degree centrality, eigenvector centrality, betweenness centrality, and eccentricity^9,19^. Prestige is defined in terms of degree prestige, rank prestige and proximity prestige^9^.

Betweenness centrality, C_B_(i), is defined as the number of times a node, i, falls on the geodesic path between two nodes, s and t, where *σ*_st_ is the total number of shortest paths from node s to node t and *σ*_st_(i) is the number of those paths that pass through node i; see Eq. (4)^19^.

4 $${C}_{B}\left(i\right)={\sum }_{s\ne i\ne i}\frac{{\sigma }_{st}(i)}{{\sigma }_{st}}$$

In other words, it is the amount of times “an actor connects pairs of other actors, who otherwise would not be able to reach one another”^20^. Betweenness centrality is based on the intermediary function of a node in the network, where a central node serves as a gatekeeper, thereby controlling the flow of resources between other nodes^19^. Closeness centrality is defined as the inverse of the sum of the distances of a node to all other nodes in a network, expressed as C_C_(v) in Eq. (5), where where d(v,t) is the length of the shortest path between vertices v and t^21^.

5 $${C}_{C}(v)=\frac{1}{{\sum }_{v}d(v,t)}$$

Closeness centrality can also be defined as a measurement of the proximity of a node to all other nodes in the network. A node is central if its distance to the other nodes in the network is short^18^.

Google’s PageRank is a query-independent algorithm that measures the static ranking of websites and is based on rank prestige^9^. PageRank^22^ is a variant of eigenvector centrality, and is based on the idea that highly ranked web pages are linked to other highly ranked web pages^6^. Eigenvector centrality, or prestige score, is a measure in graph theory that explains a node’s influence in a network^23^. The purpose of the eigenvector centrality measure is to locate the most central nodes in the network^24^. The eigenvector centrality of a node is influenced not only by the number of links that point to the node in question, but also by the prestige of the nodes connected to it^4^. Alternatively, eigenvector centrality is a recursive way to define importance in networks and represents a form of directed network in which “in-links” denote greater power^16^. Eigenvector centrality is determined by the largest characteristic eigenvalue of the adjacency matrix^21^. If the centrality of vertex i is designated as x_i_, the effect of prestige is accounted for by making x_i_ proportional to the average of the centralities of the network neighbors of i, where t is a vertex linked to vertex i; see Eq. (6).

6 $${x}_{i}={\frac{1}{\lambda }\Sigma }_{t\epsilon M(i)}$$

where λ is an eigenvalue constant and M(i) is a set of neighbors of v. If defining the vector of centralities x = (x_1_, x_2_,…), this can be rewritten in matrix form as in Eq. (7), where A is the adjacency matrix corresponding with eigenvalue λ:

7 $$\lambda x=Ax$$

Equation (7) shows that x is an eigenvector of the adjacency matrix corresponding with eigenvalue λ. Understanding the aim of the centralities to be non-negative, λ must be the largest eigenvalue of the adjacency matrix with x representing the corresponding eigenvector (according to the Perron-Frobenius theorem)^25^.

Eccentricity is a measure that represents the shortest distance, or geodesic path, from a specific node to the farthest node in the network^20^. The largest eccentricity of a network is referred to as the diameter of the network^25^. Modularity represents the number of edges, up to a multiplicative constant, that fall within groups minus the expected number of edges in an equivalent network with the edges being placed at random^26^.

Similar to the ‘salience indicator’, previous studies have considered an ‘importance index’ in an effort to highlight the nodes with the highest centrality scores in a network^6,27–29^. This was defined as the geometric (or normalized) mean of the various centrality metrics, ${\underline{x}}_{g}$, as in Eq. (8)^6,27–29^:

8 $${\underline{x}}_{g}={({\prod }_{i=1}^{n}{x}_{i})}^{\frac{1}{n}}$$

Where n is the total number of values and x_i_ (x_2_, x_1_,…, x_n_) are the individual numbers in the data set. The formula is equivalent to what is displayed in Eq. (9)^6,27–29^.

9 $$\sqrt[n]{{x}_{1}\times {x}_{2}\times ...\times {x}_{n}}$$

In other words, the geometric mean represents the nth root of the product of n values.

## Data Records

All datasets presented in this Data Descriptor are stored at Figshare (https://figshare.com/projects/Academic_Medical_Center_Network_Topology/137301). Individual datasets stored as comma separated values files are described below. The 40 AMCs and their top-level URL used for website crawling are recorded for reference^30^. The network topology datasets include information about the nodes representing web pages, internal edges representing hyperlinks between web pages within one website, and external edges representing hyperlinks between web pages of different websites^31^.

In addition, individual node-specific metrics^32^ and global network-wide metrics^33^ are reported. The mean global network-wide metrics values are reported in Table 2. For each AMC website, static^34^ and interactive^35^ visualizations of their network graphs constructed using nodes and internal edges were generated. Scatter plots and the associated plotted data^36^ were also generated to show the degree distribution of each of the 40 website network topologies.

**Table 2:** Table of the mean of each global network metric across 40 AMC websites.

Global Properties	Value
Type of network	Undirected
Nodes	1217.15
Edges	29138
Number of connected components	1
Average degree	39.059
Density	0.05299
Average path length	2.619
Diameter	4.45
Average clustering coefficient	0.3431
Assortativity	−0.5684
Modularity	0.009690
Number of communities	6.3
Reciprocity	(see Limitations)
Transitivity	0.2932

See Limitations section regarding the reciprocity metric.

Importance indices for all 40 AMC websites were analyzed. The importance index was calculated as the geometric mean of centrality metrics yielded from global network analysis, see Supplementary Table 2.

### Academic medical center top-level URLs

This dataset lists each AMC and respective website that was used as input for the web crawler. The information contained in this dataset is described in Table 3.

**Table 3:** Academic Medical Center Top-level URLs dataset description.

Column ID	Column Name	Description
A	Academic medical center	Full name of AMC
B	Top-level URL	URL of the AMC main website that was used as the starting input into the web crawler

### Academic medical center network graph data

Each AMC website has one external edges comma-separated values (CSV) dataset, one internal edges CSV dataset, and one nodes CSV dataset. These datasets are described in detail in Tables 4–6 respectively.

1. External Edges
  Hyperlinks between the AMC web pages with the same top-level URL and external websites that do not share the same top-level URL, see Table 4 for dataset description.
2. Internal Edges
  Hyperlinks between two AMC web pages with the same top-level URL, see Table 5 for dataset description. Internal edges were used to construct the network graphs that visually represent each AMC’s website structure.
3. Nodes

**Table 4:** Variables in the external edges dataset associated with each AMC website.

Column ID	Column Name	Description
A	Source	URL of the referring web page
B	Target	URL of the destination web page with a different top-level URL than the source

**Table 5:** Variables in the internal edges dataset associated with each AMC website.

Column ID	Column Name	Description
A	Source	URL of the referring web page
B	Target	URL of the destination web page with the same top-level URL as the source

**Table 6:** Variables in the nodes dataset associated with each AMC website.

Column ID	Column Name	Description
A	Crawl Depth	Number of hyperlinks between the web page and the top-level URL
B	H1-1	First H1 heading on the web page
C	H1-2	Second H1 heading on the web page
D	H2-1	First H2 heading on the web page
E	H2-2	Second H2 heading on the web page
F	Last Modified	Last date and time the web page was modified
G	Meta Description 1	First meta description on the web page
H	Meta Keywords 1	Meta keywords describing the topic of the web page
I	Title 1	First page title on the web page
J	Unique External Links	Number of unique external outlinks from the web page
K	Unique Inlinks	Number of unique internal inlinks to the web page
L	Unique Outlinks	Number of unique internal outlinks from the web page

Nodes representing each web page crawled starting from the AMC top-level URL, see Table 6 for dataset description.

### Academic medical center node-specific metrics

Each AMC website has one node-specific metrics CSV dataset. Table 7 lists descriptions of each of the node-specific centrality metrics included in these datasets.

**Table 7:** Node-specific centrality metrics associated with web pages of each AMC website.

Column ID	Column Name	Description
A	Node	Web page URL
B	Degree	Number of connections to other nodes
C	Betweenness	Measure of centrality based on the number of shortest paths between any pair of nodes in the network that pass through the node
D	Eigenvector	Measure of centrality based on the number of connections between the node and other nodes with high relative eigenvector centrality scores.
E	Closeness	Measure of centrality based on the reciprocal of the sum of the short path lengths between the node and all of the other nodes in the network
F	Eccentricity	Maximum path length between the node and all of the other nodes in the network
G	PageRank	PageRank ranks nodes in the network based on the structure of incoming and outgoing hyperlinks to the node
H	Clustering	Clustering coefficient based on the proportion of all possible triangles in the network that pass through the node

### Academic medical center network-wide metrics

**Fig. 2:**
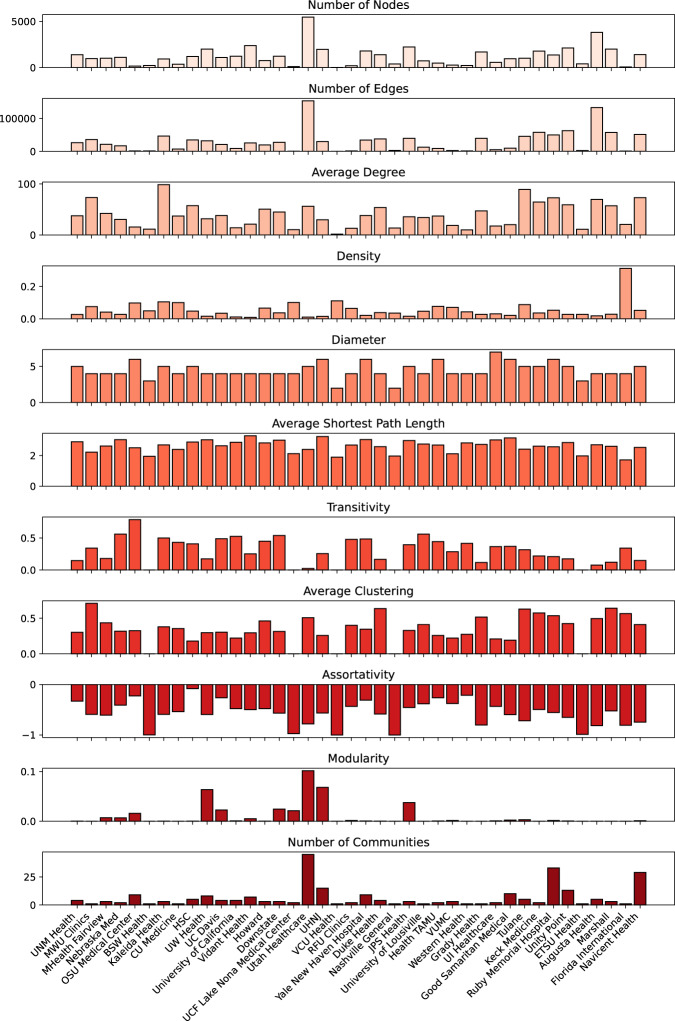
Bar graph of network-wide metric values for 40 academic medical center website network graphs. Eleven metrics are shown, including total number of nodes, total number of edges, average degree, density, diameter, average shortest path length, transitivity, average clustering, assortativity, modularity, and number of communities.

There is one CSV file that reports the network-wide metrics for each of the 40 AMCs included in this data presentation. Descriptions of these network-wide metrics are provided in Supplementary Table 3. The network-wide metric values for each of the 40 AMC websites are depicted in a bar graph format in Fig. 2. We observed a broad diversity in the 11 global network-wide metrics across the 40 different AMC website topologies included in this dataset, prompting further investigation into the functional importance of these differences on website usability.

We observed that the AMC website with relatively higher counts of nodes, representing webpages, also had relatively higher counts of edges, representing hyperlinks between these webpages. Moreover, we generally observed that the average degree of website networks were higher for websites with higher counts of nodes and edges. This suggests that websites with higher counts of webpages also have higher counts of total numbers of hyperlinks in the network and each webpage connects to a higher count of other webpages on average. In addition, we observed that diameter and average shortest path length of different AMC websites were generally similar across all website networks regardless of the number of webpages or the number of hyperlinks between webpages. Thereby, users of most AMC websites can usually expect to reach their target webpage from the website homepage through a similar number of hyperlinks. Optimizing network metrics such as diameter and average shortest path length can reduce the number of links users need to traverse between webpages, helping them find information more intuitively and faster. The detection of clusters of webpages, measured in part by network metrics such as transitivity, average clustering coefficient, assortativity, and modularity, can reveal the functional components of different AMC websites and how website structure has specialized to optimize how online users traverse different paths of hyperlinks that connect webpages to search for information. For instance, characterizing deficiencies in the connectivity and clustering of information found in webpages within an AMC website may motivate education of hospital administrators and website engineers about the importance of hyperlinks for patients to find information online and encourage them to design optimized hyperlink networks that intuitively guide patients towards information that they seek. Promotion of lesser known services offered by an AMC that otherwise might be overlooked, represented by a cluster of webpages on the AMC’s website, can be further marketed to the general public by increasing the connectivity of that cluster to other clusters of webpages within the website and important webpages with a high degree metric through the creation of new hyperlinks.

**Fig. 3:**
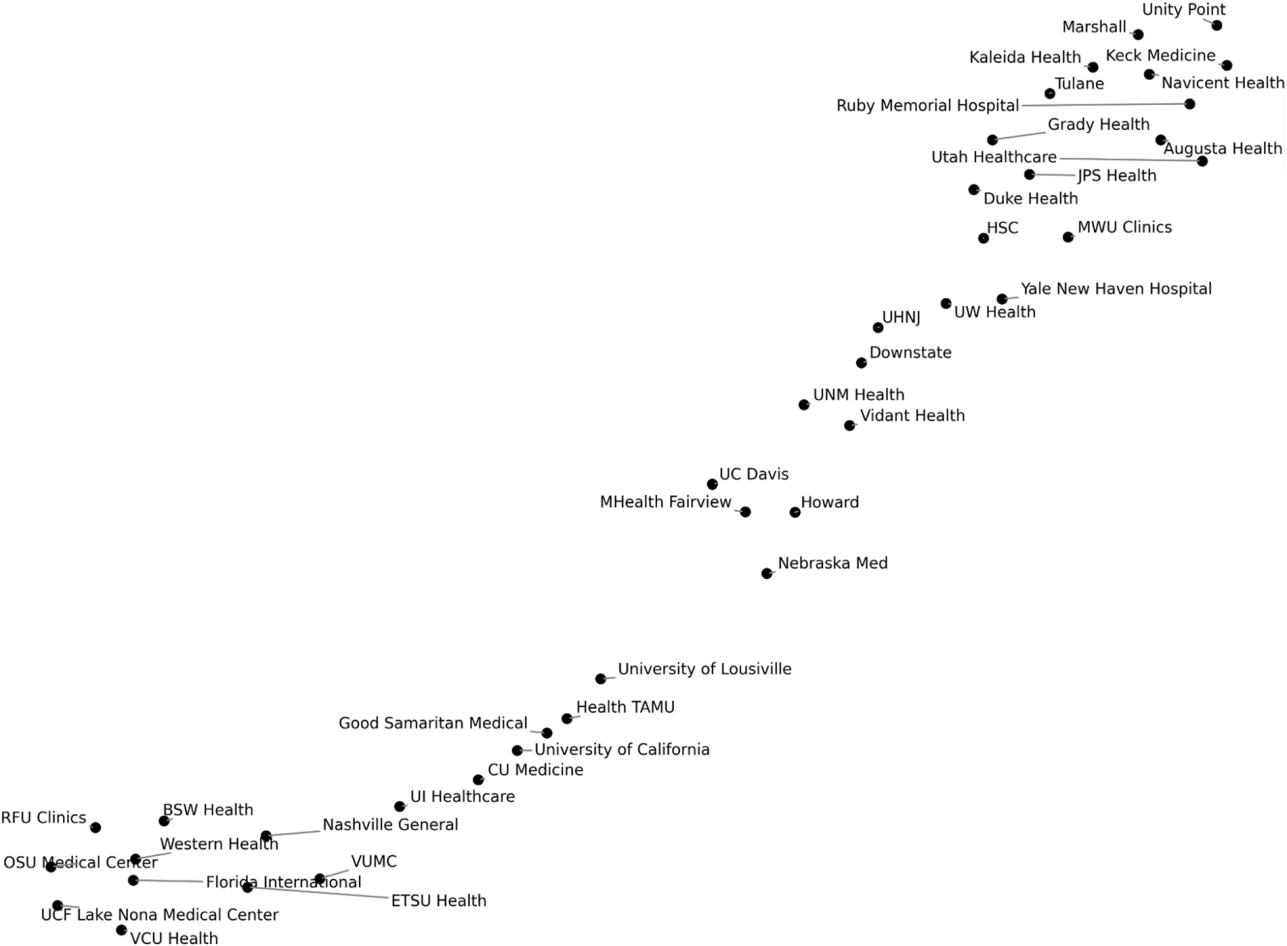
Two-dimensional uniform manifold approximation and projection plot of the eleven network-wide metrics for 40 academic medical center websites. Website networks with a more similar set of metrics values will cluster more closely.

Grouping different AMC websites by their network-wide metrics can reveal what types of website topology structures may exist and draw similarities between different website structures. To achieve this aim, Fig. 3 portrays a two-dimensional uniform manifold approximation and projection (UMAP) plot of all of the 11 global network-wide metrics described in Supplementary Table 3 for each of the 40 AMC websites. AMC websites that cluster more closely together tend to have more similar global network-wide metrics associated with website topology structure compared to other websites that do not cluster together. The UMAP plot visualizes two clusters of AMC websites distinguished by the 11 network-wide metrics that characterize the hyperlink topology of each website. For instance, we would expect that the metrics and associated hyperlink topology structure of VCU Health would be more similar to ETSU Health compared to Yale New Haven Hospital since VCU Health is closer in distance to ETSU Health on the UMAP plot. To generalize, we postulate that further clustering of academic medical websites based on global features of their hyperlink topology may yield insights into the prototypical website structures that may relate to sociocultural or functional components of AMCs across the US. Thus, further study directions can identify (1) prototypical website hyperlink topology structures of AMCs and (2) correlations between sociocultural or functional components of AMCs and features of their website hyperlink topology structure in order to guide the future purpose-built design of future AMC websites.

### Academic medical center static network visualization

**Fig. 4:**
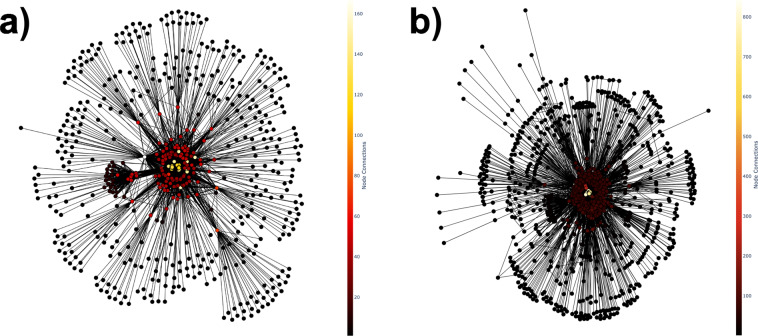
Comparison of the network graph visualization of the a) number 1-ranked academic medical center website (University of Utah Health Sciences Center; 0.495) and b) number 40-ranked academic medical center website (The University of Iowa; 0.153) based on the Importance Index. Nodes in the network are colored based on their degree, where red-colored nodes have a greater number of edges that connect to other nodes and tend to be centrally located relative to black-colored smaller sized nodes with a fewer number of connected edges.

Each AMC website has one static visual representation of the network graph structure constructed using the nodes and internal edges datasets and saved as a PNG file. Nodes are sized based on their degree, where larger nodes with higher degrees have a greater number of edges that link to other nodes. Nodes are positioned using the Fruchterman-Reingold force-directed algorithm for 50 iterations. Figure 4 provides two examples of a network topology graph visualization for two AMC websites.

### Academic medical center interactive network visualization

Each AMC website has one interactive visual representation of the network graph structure constructed using the nodes and internal edges datasets and saved as an HTML file. Nodes are colored based on their degree, where darker red nodes with higher degrees have a greater number of edges that link to other nodes. Nodes are positioned using the Fruchterman-Reingold force-directed algorithm for 100 iterations.

### Limitations

**Fig. 5:**
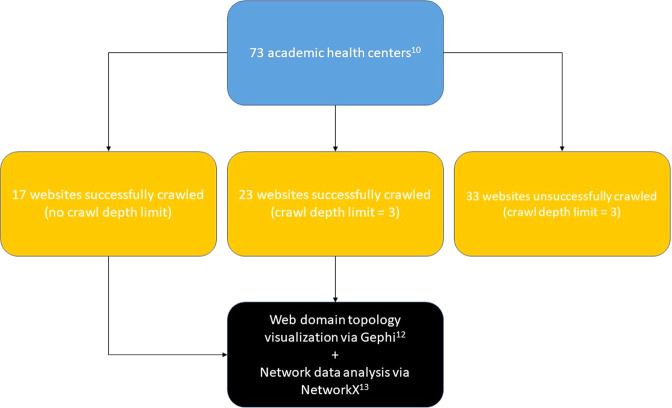
Methodology for identifying academic medical centers, compiling data for their respective websites via Screaming Frog web crawler, then visualization/analysis of data via Gephi and NetworkX. The data set consists of any website that could be crawled by a crawl depth of at least 3 or more.

Data collection via Screaming Frog web crawler was limited by two main factors. First was the intrinsic web crawler capability: for example, the respective hospital websites for the University of Oklahoma Health Sciences Center and the University of Tennessee Health Science Center were unable to be crawled due to excessive size. Second was the intrinsic data visualization capacity: quantities of crawled links that exceeded 3,000 URLs led to overwhelming of the subsequent attempted data visualization via NetworkX/Gephi. Thus, web crawls were adjusted to a limited depth of three to yield datasets that were able to be visualized: out of the 73 total AMCs websites, two were unable to be crawled (as aforementioned), 31 others were unable to be included in analysis due to excessive quantity crawled URLs, 23 websites were limited to a web crawl depth of three, leaving 17 websites that were able to be successfully crawled without a depth limit (for a detailed outline of this breakdown, please see Fig. 5) prior work has shown that pages on website depths exceeding three are less likely to be reached by users, thus, are less relevant in the context of information presentation, however this does inherently lead to limited representation of websites and domains^30^. While this study was not an exhaustive survey of hospital websites nor was it a comprehensive look into each website analyzed, future studies will look to expand the horizon of this endeavor in scope and depth of hospital websites.

With regard to the interpretation of results, further analysis of the ranking results in Table 2 was outside of the scope of this project. Future research may incorporate other metrics such as hospital size, budget for online presence and website maintenance to determine if this data is correlated with importance ranking. A reciprocity metric was not included in Table 2 given that the dataset in this project did not include directionality of edges, but rather considered them as links^5^.

For the Health Sciences Center at Prisma Health and University of Missouri-Columbia, unique URLs (uniform resource locators) to a primary teaching hospital were unable to be identified and subsequently analyzed; thus, the original website domain URL was utilized for scraping. Of note, since the time analysis was performed for this study, University of Miami, Georgetown University Medical Center, Louisiana State University Health Sciences Center - Shreveport, and The University of New Mexico Health Sciences Center are no longer AAHC members, but University of Miami and The University of New Mexico Health Sciences Center are included in this study. As well, Stony Brook University is now listed as an AAHC member but was not at the time of this analysis, and is thus excluded from this study.

## Technical Validation

Regarding the degree distribution of networks in this study, we found that the coefficient of determination (R^2^) ranged between approximately 0.52 to 0.83 for a majority of the networks, with exponents between −0.56 and −0.47 for the fitted power-law distributions. A few of the websites had a very low R^2^ value and did not demonstrate a scale-free behavior leading to a power-law distribution. Visual inspection of the networks showed it is likely that many of the degree distributions seem to be a mixture of smaller clusters of degree distributions. Each of these smaller clusters seem to follow a power-law form with different exponents. While interesting, this observation requires further investigation which is beyond the scope of the current work.

For the methodology of the paper, we focused on a subset of AMCs and ran the analysis for a period of three weeks. The bulk of the analysis involved crawling from the top-level URLs of the web page and extracting data that connected the websites to other medical websites, which mainly came in the form of links. These internal links from the websites served as the connections between nodes in the network and the outward links served as connections between the main website and other websites.

Regarding the future utility of the gathered data: we maintain that this study is a first step toward the development of deeper and more comprehensive hyperlink network topology datasets that will hold even further utility. As well, we believe that conducting a smaller analysis with a different subset of ten medical centers would be valuable, as even a smaller subset would likely have similar eigenfeatures to our larger dataset which demonstrates the validity of the approach. In addition, conducting a reverse analysis in which we use the topological data provided from the initial calculations to map the connections shown on the actual hospital websites would also serve to further demonstrate the utility of this data. Looking within the components of the analysis, we would propose doing a smaller analysis of our main dataset by dividing the medical centers into small groups of three centers. By doing the same calculations for the eigenfeatures, we would be able to calculate the differences of the subgroups relative to the large group dataset.

We would develop a positive and negative control for the comparison as well. The positive control would be the results that were previously gathered in our network analysis (Fig. 2) while the negative control would be a network analysis performed on a set of websites that did not have any connection to each other. We hypothesize the results from the positive control would show similar results to our initial network analysis and the negative control would demonstrate very low eigenvalues.

Next, we conducted a case study of community detection within the website network topology of University of Louisville Hospital website to show that subsets of web pages within one website network can be connected based on a common functional theme. Moreover, node-specific network metrics generated as part of the proposed dataset, such as the modularity metric used in this case study, can be used to recapitulate communities of thematically-related web pages.

**Fig. 6:**
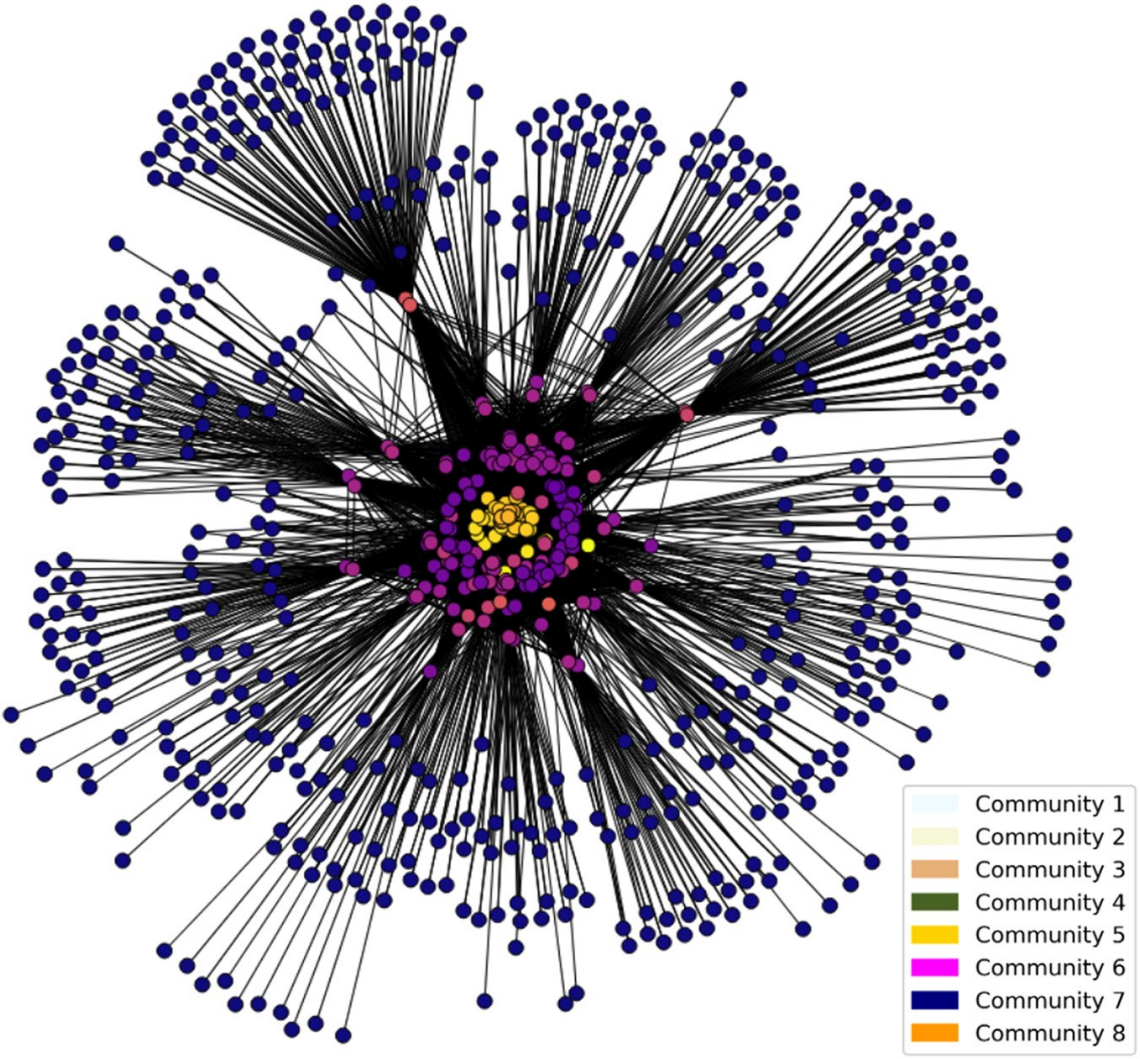
Network graph visualization of the University of Louisville academic medical center website. Community detection using Louvain clustering identified eight communities based on modularity.

From this validation case study, we detected 8 community clusters of nodes in the website topology of the University of Louisville Hospital website using Louvain clustering as shown in Fig. 6. From the 731 links on the hospital website analyzed for the validation, the cluster 0 had 63 webpages, the cluster 1 has 157 websites, the cluster 2 had 42 websites, the cluster 3 had 137 websites, the cluster 4 had 8 websites, cluster 5 had 154 websites, cluster 6 had 159 websites, and cluster 7 had 11 websites. Community clusters 0–3 had eccentricity values from 2–3 while clusters 4–7 had an eccentricity value of 4. There were little changes of eccentricity values within communities. For the overall descriptive statistics, we see that the average degree of connectedness was 636.81694, the average betweenness value was 0.01957998, the average Eigenvector value was 0.37733584, the average closeness value was 3.45765027, and the average PageRank was 0.00136612. The median degree of connectedness was 0.01388889, the median betweenness value was 0.0096276, the median Eigenvector value was 0.33227273, the median closeness value was 4, and the median PageRank was 0.0002964. For the range of values, we see that the ranges for the degree of connectedness were 0–21781.5664, the ranges for the betweenness values were 0.0003413–0.10765595, the ranges for the Eigenvector values were 0.29547292–0.59673469, the ranges for the closeness values were 2–4, and the ranges for the PageRanks were 0.00023279–0.01145176.

However, there were profile metrics in which eccentricity values across different communities were different. From cluster 0 to cluster 7, the eccentricity values rise from an average eccentricity value of 2.725 to an average of 4. Looking at the betweenness quantification, clusters 0 and 1 had values always greater than one. Conducting a t-test between cluster 0 and cluster 7, we calculated a p-value of 0.00265 in the quantification. This indicates a statistically significant difference in the quantification values between the clusters. The eigenvector values calculated generally decreased through the clusters, as the zeroth cluster had the higher average of eigenvectors while the seventh cluster had the lowest average. There were not any specific trends for PageRank or closeness values.

In network analysis, semantic grouping refers to the process of grouping webpages in a hyperlink network based on their thematic content, rather than their structural network relationships. The goal is to group nodes that have similar content together, allowing for a more meaningful representation of the data. To do so, we manually annotated different groups of webpages based on their shared themed content shown on the webpage through consensus among the co-author team as a proof-of-concept approach to grouping webpages by semantic meaning. We identified the content theme that each webpage belonged to through triangulation across multiple raters for reliability. Future research directions can aim to automate the grouping of webpages by text content using natural language processing methods. Looking at the semantic relation between web pages within each community, we observed that cluster 0 and 1 were generally not directly related to University of Louisville Hospital website links. Clusters 2 through 7 are University of Louisville Hospital sub links to different webpages of the main website, which mainly includes payment lists for procedures. Some clusters had strong semantic groupings while others were connected by more generalized themes. For example, cluster 7 generally grouped links that focused on the University of Louisville Hospital Trauma Center. Cluster 4 focused on the hospital’s financial statements and implementation strategies from different fiscal years while cluster 2 had its theme about maternal child health. Further study into how users travel across hyperlink paths between multiple web pages in each community or between different communities can reveal bottlenecks that can be optimized so that patient users can find the information they need more intuitively and conduct their search using fewer hyperlinks between different web pages.

## Usage Notes

The network data of 40 AMC websites, each consisting of nodes and edges searched to a crawl depth of 3, may be used for several downstream analyses including (1) detection of network communities^37^ and network clustering as well as (2) comparative network analysis^38^. Network communities are useful to understand how a given website is organized, how users may potentially interact with related web pages, including which web pages may receive the highest traffic and how to efficiently optimize the different paths of websites that users take to seek desired information. For instance, characterizing the different communities of web pages within a website can help AMCs optimize their websites so that patient users can find related information on different pages more intuitively. Comparative network analyses, such as network alignment, can characterize similarities and differences between different network topologies. This may be useful in identifying which types of AMC website (sub)structures exist that are common to different centers. Intuitions from such comparative analysis can help us determine which structure is expected to be most effective at delivering content to their respective user demographics when paired with website traffic data.

## Supplementary information

Supplementary Tables

## Data Availability

The code used to calculate the node-specific metrics, network-wide metrics, as well as static and interactive visualizations of each of the 40 AMC websites can be found at https://github.com/davidchen0420/Academic-Medical-Center-Topology. The Jupyter notebook AMC_Topology_Metrics.ipynb describes the steps used to calculate the metrics as comments. To run the Jupyter notebook, installation of the Anaconda distribution of Python 3.8.0+ and required scientific packages listed in the notebook is needed. Example input data and expected output results are provided in example_data.zip in the GitHub. The example input data is a subset of 3 AMC website nodes and internal edges that can also be found in the Figshare repository (see Data Records).
