# Supplementary material for: Network Analysis of Academic Medical Center Websites in the United States
Source: Sci Data. 2023 Apr 28;10:245. doi: 10.1038/s41597-023-02104-3 (PMC10147938; doi:10.1038/s41597-023-02104-3)
Supplement: Supplementary file 1 — Supplementary Tables [file 41597_2023_2104_MOESM1_ESM.pdf]

Supplementary Table 1. Excluded websites with associated reasons for exclusion.

Website	Reason for exclusion
https://www.utmedicalcenter.org/	Network security preventing crawling
https://www.ouhealth.com/	Network security preventing crawling
https://www.bonsecours.com/	Excessive domain size
https://www.emoryhealthcare.org/index.html	Excessive domain size
https://www.medstarhealth.org/	Excessive domain size
https://lluh.org/	Excessive domain size
https://www.umcno.org/	Excessive domain size
https://www.froedtert.com/	Excessive domain size
https://muschealth.org/	Excessive domain size
https://www.ohsu.edu/health	Excessive domain size
https://stanfordhealthcare.org/	Excessive domain size
https://www.upstate.edu/healthcare/	Excessive domain size
https://wexnermedical.osu.edu/	Excessive domain size
https://www.pennstatehealth.org/	Excessive domain size
https://www.bannerhealth.com/	Excessive domain size
https://www.uncmedicalcenter.org/uncmc/	Excessive domain size
https://www.utmedicalcenter.org/	Excessive domain size
https://www.universityhealthsystem.com/	Excessive domain size
https://walterreed.tricare.mil/	Excessive domain size
https://uamshealth.com/	Excessive domain size
https://www.ucihealth.org/	Excessive domain size
https://www.ucsfhealth.org/	Excessive domain size
https://www.uchealth.com/	Excessive domain size
https://hospital.uillinois.edu/	Excessive domain size
https://www.kansashealthsystem.com/	Excessive domain size
https://umiamihealth.org/	Excessive domain size
https://www.ouhealth.com/	Excessive domain size
https://www.pennmedicine.org/for-patients-and-visitors	Excessive domain size
https://www.upmc.com/	Excessive domain size

https://www.urmc.rochester.edu/strong-memorial.aspx	Excessive domain size
https://uvahealth.com/	Excessive domain size
https://www.uwmedicine.org/	Excessive domain size
https://www.wakehealth.edu/	Excessive domain size
https://www.barnesjewish.org/	Excessive domain size

Supplementary Table 2. Most influential AMCs ranked by importance index.

Rank	Importance Index	Name
1	0.495	University of Utah Health Sciences Center
2	0.462	Augusta University
3	0.455	University of Miami
4	0.454	Midwestern University
5	0.45	Tulane University Health Sciences Center
6	0.413	Marshall University
7	0.401	University of Southern California
8	0.4	West Virginia University
9	0.386	Duke University
10	0.384	University at Buffalo/The State University of New York
11	0.358	The University of Iowa
12	0.348	Emory University
13	0.305	University of Minnesota
14	0.304	Howard University
15	0.29	Virginia Commonwealth University
16	0.285	University of Colorado
17	0.282	The University of Texas Health Science Center at San Antonio
18	0.275	Mercer University Health Services
19	0.272	Yale University
20	0.26	University of Louisville
21	0.259	State University of New York Downstate Medical Center
22	0.2588	University of California, Davis
23	0.257	Medical College of Wisconsin
24	0.256	University of Wisconsin - Madison
25	0.255	University of North Texas Health Service Center at Fort Worth
26	0.248	The Texas A&M University System Health Science Center
27	0.2379	East Carolina University
28	0.2375	Vanderbilt University
29	0.233	Rutgers Biomedical and Health Sciences
30	0.23	Oklahoma State University Center for Health Sciences
31	0.223	University of Nebraska Medical Center
32	0.218	Rosalind Franklin University of Medicine and Science
33	0.197	University of Central Florida
34	0.188	Baylor College of Medicine
35	0.183	Meharry Medical College
36	0.175	East Tennessee State University
37	0.174	University of California System
38	0.166	Western University of Health Sciences
39	0.159	New York Institute of Technology
40	0.153	The University of Iowa

Supplementary Table 3. Network-wide metrics of each AMC website.

Column ID	Column Name	Description
A	Name	Academic medical center name
B	Nodes	Number of nodes in the network
C	Edges	Number of undirected edges in the network
D	Average Degree	Mean of the degree for all nodes in the network
E	Number of Connected Components	Number of nodes in the network that are connected
F	Average Shortest Path Length	Mean of the shortest path length between all possible pairs of nodes in the network
G	Density	Ratio of edges observed in the network and the maximum number of possible edges in the network
H	Diameter	The longest path length out of the shortest path lengths between all possible pairs of nodes in the network
I	Transitivity	Ratio of the number of triangles observed in the network and the number of connected triples of nodes in the network
J	Average Clustering	Mean of the clustering coefficient for each node in the network
K	Assortativity	Metric based on the similarity of connections in the graph relative to the node degree.
L	Modularity	Metric based on the network structure that measures the density of connections between nodes within the same module and between different modules
M	Number of Communities	Number of different sets of unique nodes grouped together using semi-synchronous label propagation algorithms
